# Paying to publish: A cross-sectional analysis of article processing charges and journal characteristics among 87 pathology journals

**DOI:** 10.1016/j.acpath.2024.100153

**Published:** 2024-11-20

**Authors:** Landon M. Clark, Dayle K. Wang, Brian D. Adkins, Valerie A. Fitzhugh, Philip D. Walker, Shazia S. Khan, Oluwole Fadare, Laura D. Stephens, Alice C. Coogan, Garrett S. Booth, Jeremy W. Jacobs

**Affiliations:** a Vanderbilt University School of Medicine, Nashville, TN, USA; b Department of Pathology, University of Texas Southwestern Medical Center, Dallas, TX, USA; c Department of Pathology, Immunology and Laboratory Medicine, Rutgers New Jersey Medical School, Newark, NJ, USA; d Department of Pathology and Laboratory Medicine, Rutgers Robert Wood Johnson Medical School, New Brunswick, NJ, USA; e Pathology Service Line, Robert Wood Johnson Barnabas Health, West Orange, NJ, USA; f Annette and Irwin Eskind Family Biomedical Library and Learning Center, Vanderbilt University, Nashville, TN, USA; g Departments of Pathology and Laboratory Medicine, Yale School of Medicine, New Haven, CT, USA; h Department of Pathology, University of California San Diego, La Jolla, CA, USA; i Department of Pathology, Microbiology, and Immunology, Vanderbilt University Medical Center, Nashville, TN, USA; j Department of Laboratory Medicine and Pathology, Mayo Clinic, Rochester, MN, USA

**Keywords:** Academic medicine, Article processing charges, Biomedical publishing, Inequity, Open access, Pathology, Publishing model, Publishing fees

## Abstract

Article processing charges are increasingly being levied on authors via publication fees to provide open access to readers. These charges may impose challenges to early career physicians seeking to publish research but pathology journal article processing charges have not been investigated to date. We aimed to quantify pathology journal article processing charges and investigate the potential associated factors. We performed a cross-sectional analysis of manuscript article processing charges among the 87 pathology journals in the “Pathology” category in Journal Citation Reports (Clarivate) and associated journal variables: publication model (open access vs hybrid), impact factor, year founded, journal location, journal publisher, medical society affiliation, whether the journal is published in print vs online, and subscription cost to institutions. Most (70.1 %, 61/87) journals were hybrid, while 29.9 % (26/87) were open access. Overall, the median cost to publish open access was significantly greater for hybrid journals compared with open access journals ($3710 vs $1735; *P*<0.0001). Article processing charges positively correlated with impact factor, journal publisher, and institutional journal subscription costs in bivariate analysis. In multivariable analysis, impact factor associated with higher charges, whereas open access journals, medical society affiliation, and location in a European country outside of the United Kingdom were associated with lower charges. There was no significant association between publication frequency, years since journal founding, or print and online publication. Understanding the potential fees that may impact pathologists attempting to publish in the biomedical literature as well as the options for covering these costs is crucial to ensure equitable career advancement opportunities.

## Introduction

Academic productivity can be assessed by various metrics, including publication of peer-reviewed manuscripts. The publication and distribution of scientific work allow for dissemination of new knowledge and constitute a driving force for career advancement.^1^ These factors have contributed to the increase in published literature with annual growth rates estimated to be 5 % or more by some models.^2^ The primary research articles published in open access (OA) models account for a significant proportion of this expansion, having doubled from 2006 to 2010 with an estimated 30–55 % of articles currently published in OA.^3^, ^4^, ^5^

The increase in OA publications is partially linked with emerging requirements by funding sources, including the National Institutes of Health and other international agencies, which are intended to ensure public access to taxpayer-funded research.^6^^,^^7^ Rather than levying fees on readers or institutions for article access (i.e., traditional subscription-based journals), OA shifts these fees to authors in the form of article processing charges (APCs). OA articles can be published by journals that are completely OA or by hybrid journals that offer a choice between subscription-only and OA journals (Table 1).^8^, ^9^, ^10^, ^11^, ^12^, ^13^, ^14^, ^15^, ^16^ APCs are generally instituted for OA submissions regardless of whether the journal is OA or hybrid, but they may be subsidized or discounted based on individual OA agreements that are individually negotiated between various publishers and institutions.^17^^,^^18^

**Table 1:** Overview of journal publishing models.

	Definition	Details
**Open access**	Completely open access. All articles are published freely accessible to readers without subscription fees	-Citation advantage^8^^,^^9^ -Increased public accessibility^9^^,^^10^ -May be newer journals/lower impact factors^11^ -Subset of OA journals employ “predatory” practices by having poor peer review processes^12^^,^^13^
**Hybrid**	Mixed open access and subscription; readers can access select articles without subscription fees, depending upon how the authors published the manuscript	-Generally higher APCs than OA journals^14^^,^^15^ -Charge both APCs and subscription fees^14^
**Subscription**	Completely subscription-based; readers can access articles only with subscription fees	-Authors are usually not charged for publication but readers pay subscription fees^16^ -Articles are less available and more difficult to access^9^

APCs: article processing charges; OA: open access

The proponents of OA models often reference the higher citation rate for OA articles and their ability to rapidly disseminate scientific knowledge without paywalls or subscription fees.^10^^,^^15^^,^^19^, ^20^, ^21^ This was evident during the COVID-19 pandemic, in which OA platforms, including OA preprint servers such as BioRxiv and MedRxiv, were crucial for accelerating COVID-19 research.^9^^,^^10^^,^^15^^,^^19^^,^^22^^,^^23^ Additional benefits of OA publishing include author retention of copyright and the flexibility allowed by particular licenses compared with copyright agreements including added flexibility to immediately use figures that the author(s) may have created. When an author retains the copyright, permission from and/or payment to the journal is not required to use their work.

Others have criticized OA models due to their APCs, which may present a financial barrier to publication, especially for trainees and early career physicians and other healthcare practitioners.^11^^,^^24^, ^25^, ^26^, ^27^ APCs may also perpetuate existing inequities within medical fields, with disproportionate impacts on researchers from historically excluded groups.^24^^,^^28^, ^29^, ^30^ The previous findings have demonstrated that the subsidization of APCs correlates with an increased number of OA publications,^31^ which further supports the possibility that APCs are a financial disincentive for publishing OA studies. As OA journal articles have greater audience reach, early career physicians and/or individuals with limited research funds may rely on paying these APCs “out-of-pocket” to achieve academic recognition. Additionally, despite the increasing numbers of women physicians entering academic medicine, including pathology, bibliometric studies continue to demonstrate gender imbalances in publication output and influence.^32^, ^33^, ^34^, ^35^, ^36^, ^37^, ^38^ The degree to which, if any, these APCs influence publication differences by gender has not been established; however, no single factor can account for academic success, be it publications, award conferral, or academic rank promotion. Academic pathology departments should be cognizant of the financial burden APCs place on faculty, particularly for non-grant funded studies, and special consideration for equitable payment and reimbursement of APCs is warranted.

APCs have been shown to influence citation rates, but may be much more likely to influence the submission practices of individual faculty members, particularly if not covered through grant funding or other institutional subsidies. However, APC details among pathology journals specifically are unknown. Given that early career pathologists at academic institutions have reported self-funding as much as 86 % of their publications,^1^ we aimed to quantify pathology journal APCs and explored, identified, and categorized journal characteristics that may influence the APCs. These findings may aid additional studies on how these financial barriers could contribute to inequities within the field.

## Materials and methods

The objectives of this study were to quantify pathology journal APCs by journal publication model— OA, subscription-based, or hybrid (Table 1)—and assess various journal characteristics to evaluate whether they were associated with the APC amount.

### Journal list

We assessed all journals in the “Pathology” category using the Journal Citation Reports (JCRs) from Clarivate (n = 87 journals).^39^ The complete journal list is available in Supplemental Table 1. The APCs for the manuscript categories “original research” and “review” were collected from each journal's website in March 2024, if available. All charges are in United States Dollars (USD). If the APC in USD was not available, we used the Fiscal Data Currency Exchange Rates Converter provided by the US Government Department of Treasury (https://fiscaldata.treasury.gov/currency-exchange-rates-converter/). If the APC differed between society members and non-members, the non-member rate was used. We did not include submission fees or charges for color figures. We also did not include any potential taxes, including value-added tax, goods and services tax (GST), or other sales taxes. Likewise, if the APC differed by country of the submitting author (i.e., through a waiver or subsidization program), we used the APC charged to authors in the US for standardization purposes, though we did assess whether information regarding a potential APC waiver/subsidization was readily available on each journal's respective website.

For hybrid journals, we obtained the APC levied for publishing OA. The journal variables that were recorded, if available, included the 2022 journal impact factor (IF) obtained from JCR, the year the journal was founded, the country in which the journal was located, and the country's income group based on the World Bank classification,^40^ the journal publisher, if the journal's website stated an affiliation with a medical society (yes/no), the publication frequency (issues/year), whether the journal is published in print, and the subscription cost for access to the online and/or print version of the journal as of April 2024 via Ulrichsweb (ProQuest, Ann Arbor, MI, USA).

### Data analysis

All statistical analyses were performed using Prism (version 10.2.3, GraphPad Software, USA) or EasyMedStat (v.3.30, France). Normality was assessed with the Shapiro–Wilk test. For normally distributed data, unpaired two-sided *t*-tests and one- or two-way ANOVAs with multiple comparisons were used. For variables that were not normally distributed, the Mann–Whitney and Kruskal–Wallis tests were used. To assess for correlation between journal IF and APC, Pearson correlation coefficient was used for normally distributed data and Spearman's rank correlation coefficient was used for non-normally distributed data. Simple linear regression models were produced for visual depiction. Medians and interquartile ranges (IQRs) were reported for non-normally distributed data. *P*-values <0.05 were considered statistically significant.

A multivariable linear regression was performed to assess the relation between journal APC and explanatory variables: 2022 IF; years since journal founding; publication frequency (issues/year), medical society affiliation; publishing model; whether the journal was published in print; and journal country [organized by US, United Kingdom (UK), Europe (non-UK), and others]. The lack of available subscription costs for online and print access for a subset of the journals precluded our ability to include this variable in the multivariable analysis, though we did assess it in the bivariate analysis. Data were checked for multicollinearity with the Belsley–Kuh–Welsch technique. Heteroskedasticity and normality of residuals were assessed respectively by the Breusch–Pagan test and the Shapiro–Wilk test. A *P*-value <0.05 was considered statistically significant. The Newey West correction for heteroskedasticity was applied.

As all data were publicly available and no protected or identifiable information were obtained in accordance with the Code of Federal Regulations (CFRs), 45 CFR 46.102; this study was deemed exempt from ethical review by the Mayo Clinic Institutional Review Board.

## Results

Among 87 pathology journals analyzed, 70.1 % (61/87) were hybrid and 29.9 % (26/87) were OA. No journals were completely subscription-based. Most journals reported an APC for each manuscript category (research, n = 82; review, n = 83). Three journals do not publish research, two do not publish review articles, one only provided cost per page, and one did provide the APC. Among the 80 journals that reported an APC >0$ for at least one manuscript type, 78.8 % (63/80) of journal websites referenced a potential APC waiver/subsidization program. However, the requirements for these programs and the information available to authors differed substantially among journals. The journal details are depicted in Table 2.

**Table 2:** Variables for the 87 journals included in the study.

Variable	Median (IQR) or n (%)
Journal impact factor	2.4 (1.3–4.1)

Journal location	
USA	38 (43.7 %)
England	16 (18.4 %)
Germany	5 (5.7 %)
Japan	5 (5.7 %)
Denmark	3 (3.4 %)
Netherlands	3 (3.4 %)
Switzerland	3 (3.4 %)
France	2 (2.3 %)
Italy	2 (2.3 %)
Poland	2 (2.3 %)
Australia	1 (1.1 %)
India	1 (1.1 %)
Malaysia	1 (1.1 %)
New Zealand	1 (1.1 %)
South Korea	1 (1.1 %)
Spain	1 (1.1 %)
Turkey	1 (1.1 %)
Ukraine	1 (1.1 %)

Year of founding	1986 (1968–1997)

Publication frequency (issues/year)	
1	9 (10.3 %)
3	3 (3.4 %)
4	17 (19.5 %)
6	28 (32.2 %)
7	2 (2.3 %)
8	1 (1.1 %)
10	3 (3.4 %)
12	24 (27.6 %)

Publisher	
Elsevier	16 (18.4 %)
Wiley	16 (18.4 %)
Springer	9 (10.3 %)
Lippincott Williams & Wilkins	7 (8.0 %)
Sage Publications Inc	6 (6.9 %)
Taylor & Francis	3 (3.4 %)
Hindawi	2 (2.3 %)
Humana Press Inc	2 (2.3 %)
Karger	2 (2.3 %)
Oxford University Press	2 (2.3 %)
Annual Reviews	1 (1.1 %)
BMC	1 (1.1 %)
BMJ Publishing	1 (1.1 %)
College of American Pathologists	1 (1.1 %)
Company Biologists	1 (1.1 %)
Dove Medical Press	1 (1.1 %)
Dustri-Verlag Dr Karl Feistle	1 (1.1 %)
E-Century Publishing Corp	1 (1.1 %)
F Hernandez	1 (1.1 %)
Federation Turkish Pathology Soc	1 (1.1 %)
Frontiers Media SA	1 (1.1 %)
Japanese Society of Toxicologic Pathology	1 (1.1 %)
Korean Society of Pathologists	1 (1.1 %)
Lepra	1 (1.1 %)
Malaysian Journal Pathology	1 (1.1 %)
Masson Editeur	1 (1.1 %)
Pacini Editore	1 (1.1 %)
Scientific Scholar	1 (1.1 %)
Termedia Publishing House	1 (1.1 %)
Vesalius Univ Medical Publications	1 (1.1 %)
Wolters Kluwer Medknow Publications	1 (1.1 %)
Zaporizhzhya State Medical University	1 (1.1 %)

Publication model	
Hybrid	61 (70.1 %)
Open access	26 (29.9 %)

Medical society affiliation	
Yes	58 (66.7 %)
No	28 (32.2 %)
Unknown	1 (1.1 %)

Publication access	
Both print and online	76 (87.4 %)
Online only	7 (8.0 %)
Print only	2 (2.3 %)
Unknown	2 (2.3 %)

### Manuscript category and journal publication model

Among all journals combined, there was no significant difference in the median APC between manuscript categories {research: $3487 [interquartile range (IQR): $2090–$3992], 95 % confidence interval (CI): $2970-$3710 vs review: $3473 ($2060-$3990), 95 % CI: $2970-$3650; *P* = 0.85}. Similarly, there was no difference in the median APC between the two manuscript categories in either hybrid journals or OA journals (Table 3).

**Table 3:** Comparison of article processing charge (APC) between manuscript types for open access journals, hybrid journals, and all journals combined.

	Open access			Hybrid			All journals		
	Median (IQR)	95 % CI	*P*-value	Median (IQR)	95 % CI	*P*-value	Median (IQR)	95 % CI	*P*-value
**Research**	$1758 ($218– $2645)	$272–$2630	0.67	$3715 ($3278–$4225)	$3590–$3950	0.94	$3487 ($2090–$3992)	$2970–$3710	0.85
**Review**	$1703 ($79– $2598)	$200–$2500		$3710 ($3290–$4190)	$3590–$3950		$3473 ($2060–$3990)	$2970–$3650	

Note: All APCs are in US Dollars. Abbreviations: IQR, interquartile range; CI, confidence interval.

**Fig. 1:**
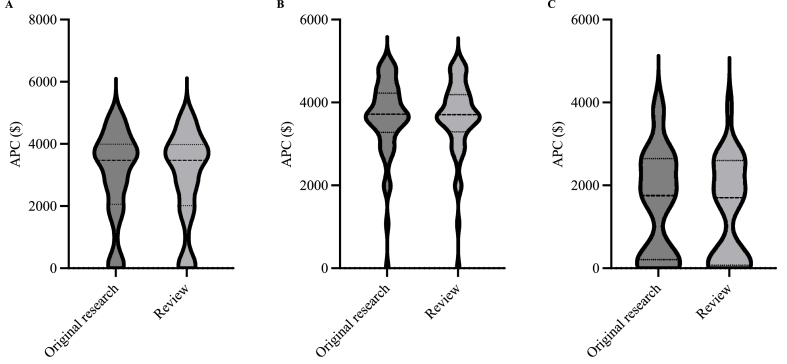
Comparison of article processing charge (APC) by manuscript category among A) all journals, B) hybrid journals, and C) open access journals.

An APC was reported for at least one manuscript category in 98.4 % (60/61) of hybrid journals and 92.3 % (24/26) of OA journals. Regarding individual manuscript categories, 95.1 % (58/61) of hybrid journals and 92.3 % (24/26) of OA journals reported an APC for research, while 96.7 % (59/61) of hybrid journals and 92.3 % (24/26) of OA journals reported an APC for reviews. The median cost to publish OA was significantly greater for hybrid journals compared with OA journals for both review and research manuscripts (Fig. 1, Table 4).

**Table 4:** Article processing charge (APC) by manuscript category between open access and hybrid journals.

Manuscript category	Open access		Hybrid		*P*-value
	Median (IQR)	95 % CI	Median (IQR)	95 % CI	
All categories combined	$1735 ($200–$2630)	$380–$2060	$3710 ($3290–$4190)	$3610–$3850	<0.0001
Research	$1758 ($218–$2645)	$272–$2630	$3715 ($3278–$4225)	$3590–$3950	<0.0001
Review	$1703 ($79–$2598)	$200–$2500	$3710 ($3290–$4190)	$3590–$3950	<0.0001

Abbreviations: IQR, interquartile range; CI, confidence interval.

### Journal publisher

The 87 journals were published by 32 unique publishers, with 62.1 % (54/87) published by one of the five publishers. The most common publishers were Wiley (n = 16) and Elsevier (n = 16). Among these 54 journals and 5 publishers, 88.9 % (48/54) were hybrid journals and 11.1 % (6/54) were OA journals. Given the small number of OA journals for any single publisher, we analyzed the median APC for manuscripts in both hybrid and OA journals combined among these five publishers.

There were statistically significant differences in the APCs among the publishers for all manuscript categories combined (*P* = 0.0007) and for research manuscripts only (*P* = 0.036), but no statistically significant difference was observed among publishers when the APCs for only reviews were assessed (Table 5). For all manuscript categories combined, the highest median APCs were for journals published by Springer [$3990 ($3590– $4390)], whereas journals published by Elsevier [$3000 ($2100– $3560)] had the lowest median APCs (Table 5).

**Table 5:** Article process charge (APC) by manuscript category for open access and hybrid journals combined among the five most common publishers.

Publisher	All manuscript categories		Research		Review	
	Median (IQR)	95 %% CI	Median (IQR)	95 %% CI	Median (IQR)	95 %% CI
Elsevier	$3000 ($2100–$3560)	$2570–$3500	$3000 ($2100–$3560)	$2100–$3560	$3120 ($2218–$3680)	$2100–$3720
LWW	$3473 ($2806–$3997)	$2806–$3997	$3473 ($2806–$4105)	$2806–$4429	$3473 ($2806–$4105)	$2806–$4429
Sage	$3735 ($2650–$3950)	$2650–$3950	$3735 ($2650–$3950)	$2650–$3950	$3735 ($2650–$3950)	$2650–$3950
Springer	$3990 ($3590–$4390)	$3590–$4190	$4090 ($3615–$4490)	$3290–$4790	$3990 ($3590–$4390)	$3590–$4590
Wiley	$3730 ($3453–$4330)	$3610–$4330	$3730 ($3453–$4330)	$3400–$4330	$3730 ($3453–$4330)	$3400–$4330

Abbreviations: IQR, interquartile range; CI, confidence interval; LWW, Lippincott Williams & Wilkins.

### Journal geographic location

The 87 journals were based in 18 unique countries, with 43.7 % (38/87) in the US. Four countries accounted for 73.6 % (64/87) of all journals [US; England (n = 16); Germany (n = 5); and Japan (n = 5)]. Two lower-middle-income countries (India and Ukraine) were represented, with 2.3 % (2/87) of journals affiliated with these countries. There were no journals affiliated with countries classified as low-income. Hybrid journals were more likely than OA journals to be based in the US (52.5 %, 32/61 vs 23.1 %, 6/26; χ^2^ = 6.40; *P* *=* 0.011).

Analysis of APCs by manuscript category and region revealed that, for all publication models combined, the highest APCs for all manuscript categories were for journals in the US [$3620 ($2806–$3997)], but the difference in the median APCs among journals in the US, Europe, and Asia was not statistically different (*P* = 0.082) (Table 6). Similar findings were observed when specifically assessing APCs for research manuscripts and reviews. When the analysis was limited to only hybrid journals and only OA journals, there was again no statistical difference in the median APC among journals in the US, Europe, or Asia for all manuscript categories combined, or for research and reviews.

**Table 6:** Analysis of article processing charge (APC) by manuscript category, journal type, and region.

Open access							
Manuscript category	United States		Europe		Asia		*P*-value (difference in median APCs among regions)
	Median (IQR)	95 % CI	Median (IQR)	95 % CI	Median (IQR)	95 % CI	
All categories	$1780 ($1125–$1963)	$0–$2000	$1898 ($214–$2663)	$380–$2650	$350 ($50–$2098)	$0–$2630	0.24
Research	$1780 ($750–$1975)	$0–$2000	$2060 ($272–$2700)	$272–$2700	$350 ($50–$2098)	$0–$2630	0.37
Review	$1780 ($750–$1975)	$0–$2000	$1735 ($38–$2650)	$38–$2650	$350 ($50–$2098)	$0–$2630	0.62

Hybrid							

Manuscript category	United States		Europe		Asia		*P*-value (difference in median APCs among regions)

Median (IQR)	95 % CI	Median (IQR)	95 % CI	Median (IQR)	95 % CI
All categories	$3670 ($3380–$4163)	$3500–$3950	$3730 ($3240–$4550)	$3590–$4128	$3800 ($3333–$4140)	$3240–$4190	0.80
Research	$3670 ($3358–$4225)	$3473–$3950	$3730 ($3240–$4550)	$3400–$4330	$3800 ($3333–$4140)	$3240–$4190	0.89
Review	$3670 ($3358–$4108)	$3473–$3950	$3725 ($3280–$4495)	$3400–$4330	$3800 ($3333–$4140)	$3240–$4190	0.90

All journals							

Manuscript category	United States		Europe		Asia		*P*-value (difference in median APCs among regions)

Median (IQR)	95 % CI	Median (IQR)	95 % CI	Median (IQR)	95 % CI
All categories	$3620 ($2806–$3997)	$3473–$3720	$3240 ($1940–$4070)	$2650–$3710	$2935 ($275–$3895)	$200–$3990	0.082
Research	$3620 ($2806–$3997)	$3290–$3750	$3320 ($1955–$4085)	$2500–$3750	$2935 ($275–$3895)	$0–$4190	0.30
Review	$3620 ($2806–$3997)	$3290–$3750	$3240 ($1910–$4070)	$2100–$3730	$2935 ($275–$3895)	$0–$4190	0.27

Abbreviations: IQR, interquartile range; CI, confidence interval.

### Journal impact factor

Journal IF was available for all journals. There was no significant difference in the median IF between hybrid and OA journals [2.8 (1.6–4.8), 95 % CI: 2.0–3.4 vs 2.0 (1.0–3.5), 95 % CI: 1.0–3.2; *P* = 0.057]. Notably, the IF of one hybrid journal was >3 standard deviations above the mean for all journals, and therefore this journal was excluded from further bivariate IF analysis. With removal of this journal, the difference in the median IF between hybrid and OA journals remained statistically nonsignificant.

**Fig. 2:**
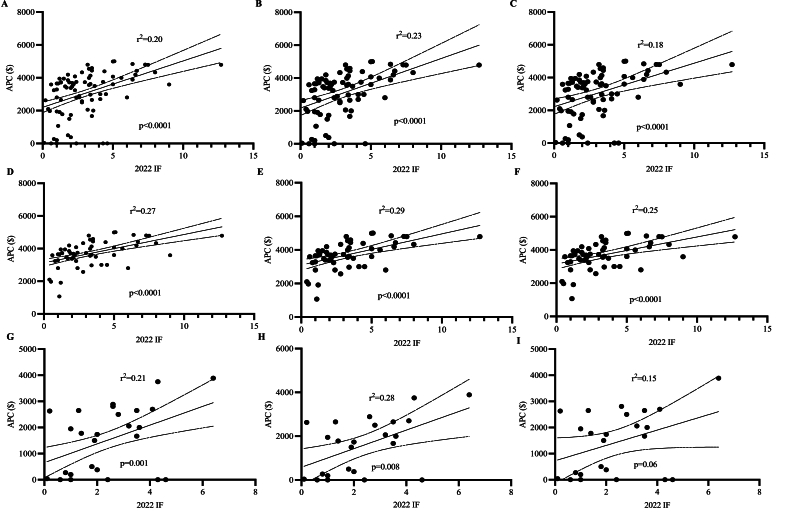
Simple linear regression models of journal impact factor (IF) vs article processing charge (APC) for A) all manuscript categories among all journals, B) research manuscripts among all journals, C) review manuscripts among all journals, D) all manuscript categories among hybrid journals, E) research manuscripts among hybrid journals, F) review manuscripts among hybrid journals, G) all manuscript categories among open access journals, H) research manuscripts among open access journals, and I) review manuscripts among open access journals.

In simple linear regression, there was a significant correlation between journal IF and APCs for all manuscript types in both hybrid and OA publication models combined, as well as individually. Similar results were observed for research manuscripts. APCs generally correlated with IF and hybrid journal type (Fig. 2). Of note, APCs were lowest among online-only journals that specialize in review articles. These journals also tended to be lower impact.

### Subscription model and subscription cost

The median number of issues published annually was significantly greater for hybrid journals compared with OA journals [6 issues/year (6–12), 95 % CI: 6–10 vs 4 issues/year (1–6), 95 % CI: 1–4; *P* < 0.0001). The APC significantly correlated with the number of annual issues among hybrid and OA journals combined and among hybrid journals only for all manuscripts, research manuscripts, and review manuscripts. No correlation was seen in OA journals between the APC and the annual number of issues for any manuscript type.

A significantly greater proportion of OA journals were published online-only compared with hybrid journals (5/24 vs 2/61; χ^2^ = 7.02; *P* = 0.008). The median APC for journals published online-only was significantly lower than the median APC for journals published in both print and online [$2630 ($1780–$2700) 95 % CI: $1780–$2700 vs $3600 ($2800–$4070) 95 % CI: $3473–$3710; *P* *=* 0.003].

As most journals were published both online and in print (87.4 %, 76/87), we assessed the annual subscription cost to institutions for online access and for print access to this group of journals, and evaluated whether APCs correlated with these subscription costs. Among the 76 journals published in both print and online, 52 (hybrid: n = 46, OA: n = 6) had a subscription price for print access, while 36 (hybrid: n = 36 hybrid, OA: n = 0) had a subscription price for online access. The median print subscription price [$1695 ($1001–$2711), 95 % CI: $1294–$2191] was not significantly different from the median online subscription price [$1684 ($918–$3149), 95 % CI: $1201–$2533; *P* = 0.94]. APCs positively correlated with both print and online subscription fees for all manuscript categories.

### Multivariable analysis

In multivariable analysis, the 2022 journal IF was associated with higher APCs (*P* < 0.0001). Conversely, OA journals (compared with hybrid journals; *P* < 0.0001), journals affiliated with a medical society (compared with those without a society affiliation; *P* = 0.001), and journals located in non-UK European country (compared with the USA; *P =* 0.03) were associated with lower APCs (Table 7). There was no significant association between publication frequency (i.e., the number of issues per year; *P =* 0.48), years since journal founding (i.e., age of the journal; *P =* 0.73), whether the journal was published in print and online (compared with online-only; *P* = 0.25), or if the journal was located in the UK (compared with the USA; *P =* 0.21) or outside of Europe (compared with the USA; *P* *=* 0.30).

**Table 7:** Multivariable analysis of APCs for all research and review manuscripts among all journals.

Variable	β coefficient (95 % CI)	*P*-value
Intercept	3567 [324; 3929]	<0.0001
2022 impact factor	204 [144; 263]	<0.0001
Country (reference: USA)		
UK	241 [−133; 615]	0.21
Europe (non-UK)	−347 [−661; −34]	0.03
Other	−204 [−596; 187]	0.30
Years since founding	−1 [−8; 5]	0.73
Publication frequency (issues/year)	−17 [−63; 30]	0.48
Publishing model (reference: hybrid)		
Open access	−2171 [−2581; −1761]	<0.0001
Medical society affiliation	−404 [−642; −166]	0.001
Published in print (reference: Print and online or print only)		
Online-only or not available	259 [−180; 697]	0.25

## Discussion

This systematic investigation of journal APCs across 87 pathology journals demonstrated that hybrid journals charge a significantly higher APC than OA journals, paralleling findings from other medical specialties, such as oncology.^28^ Additionally, bivariate analysis revealed that pathology journal APCs positively correlate with several variables including journal IF, journal publisher, and institutional journal subscription costs. APCs also positively correlated with the number of journal issues published annually among hybrid journals, but not among OA journals, though it is important to note that a significantly greater proportion of pathology OA journals published online-only, which may influence this finding. As such, publication frequency was not significant in multivariable analysis.

We also found that, while not statistically significant, pathology journals in the US had the highest APCs among all countries, and APCs for journals in the US were significantly higher than journals in Europe outside of the UK. These findings mirror other specialties with prior studies demonstrating that oncology journals from North America have the highest APCs,^28^ and an analysis of 97 gastroenterology journals found that journals based in the US/UK have higher APCs compared with non-US/UK journals.^41^ Journal IF has also been shown to positively correlate with APCs in other medical fields, including oncology,^21^^,^^28^ illustrating that publishers play into the demand and reputation of journals.

Inequities persist within the healthcare industry and in pathology specifically. As academic productivity and bibliometrics (e.g., Hirsch index) are often considered requisite for career promotion,^28^^,^^30^ APCs may contribute to these inequities. Fees may disproportionately affect those with less funding and those early in their careers by hindering their ability to achieve highly cited publications necessary for advancing in rank. These individuals often must resort to self-funding their publication,^1^ highlighting the challenges inherent to the “publish to promote” culture. As journal APCs have become a mainstay in modern publishing landscapes, these fees may represent significant financial barriers for researchers. For example, one study found that a median of 7 (range 2–19) first and senior authored papers were necessary for a non-tenure track pathologist to advance from assistant to associate professor.^1^ In a hypothetical scenario, with a median APC of approximately $3710 to publish OA in hybrid journals, and median APC of $1735 to publish in OA journals, an assistant professor pathologist would incur anywhere from $3470 to $70,490 if they choose, or are mandated, to publish OA.

Additionally, while some academic institutions may provide subsidized or negotiated APCs for particular publishers, thereby advancing researchers’ careers, this could inadvertently contribute to disparities if these funding/subsidization agreements are not equitably distributed. For example, the Association of American Medical Colleges (AAMC) reported that there were 1999 assistant professors in pathology in 2023.^42^ If 1999 individuals published 7 manuscripts either in an OA journal or as OA in a hybrid journal, the costs would be between $24,277,855 and $48,555,710. Thus, it could cost one cohort of assistant professor pathologists almost $50 million to advance to associate professor. As some pathology departments and institutions may be more likely to provide funding or cover these costs, the pathologists at these institutions will receive this inherent advantage. The development of a department-level system that supports faculty in meeting these publishing expenses may be a worthwhile investment by pathology leadership across institutions.

Furthermore, many junior faculty members may be unaware of funding/subsidization agreements and the role that these agreements might play in their future publishing endeavors. Better education and awareness regarding differences in APCs across journal types and subsidization models could help researchers avoid unexpected publishing costs. In the context of academic medical centers, junior faculty or pathology departments are encouraged to contact their biomedical libraries to inquire about their participation in OA or transformative agreements with publishers.^18^ Nevertheless, at present, researchers do have some alternative publishing options and avenues to minimize costs. For instance, many pathology journals in this study (70 %) are hybrid journals, and therefore have a non-OA publishing option, which allows researchers to publish their work with minimal to no fees. However, there is growing support among countries and international funding coalitions for fully OA publishing models.^43^^,^^44^ Thus, both the proportion of pathology journals available to publish in without OA fees and the proportion of individuals that are “allowed” to publish their research as non-OA may decrease. Notably, we found that it was significantly cheaper to publish OA research in OA journals compared with OA in hybrid journals, suggesting that these fully OA journals may be an attractive alternative to researchers electing (or required) to publish OA research while minimizing costs. An additional potential consideration could be cost-sharing between the institutions representing the first and senior authors.

Methods to help mitigate the financial barriers that APCs may represent for OA publishing have been proposed. These include agreements between institutions and publishers, collective funding partnerships among multiple institutions, and special allotments in funding for APCs.^17^^,^^18^^,^^45^ APC waivers are also occasionally provided by publishers, but the requirements for these waivers may not be readily available on the journal website or the waiver details may not be made available if/until the manuscript is accepted. As such, authors may not even know whether they qualify for or will receive a discounted APC at the time of submission. Furthermore, while most journals do mention the potential availability of subsidized or waived APCs in their “author information” sections, many journal websites direct authors to consult their institution or other body to determine if there is an agreement in place with the publisher, or alternatively to inquire with and provide personal information to the publisher directly to determine eligibility. In addition to these considerations, the likelihood of a waiver decreases with increasing APCs.^28^^,^^45^ Nevertheless, there has recently been an increase in the availability of award-based waivers and publisher-based discounts for individuals located in low- and lower-middle-income countries, which may serve to reduce barriers to research and biomedical publishing for authors in these geographic regions.^30^^,^^46^, ^47^, ^48^, ^49^

It is important to consider additional strategies to reduce the financial barriers in publishing OA and how these methods facilitate dissemination of scientific knowledge.^28^ One potential solution might involve fine-tuning of the one-sized APC publishing model, wherein APCs differ by various characteristics of the submitting author, including grant funding availability, number of years in practice, or publication record, in addition to geographic variables. Additional solutions might involve subsidization or support for those in training and those without grant or faculty funds. Finally, medical specialty associations and professional medical societies represent an important avenue by which to support their members in research, which could be achieved with the subsidization of APCs and research costs in general. For example, the Cytopathology Foundation (CF)^50^ introduced a model to support OA by subsidizing APCs for publishing in cytopathology journals [e.g., CytoJournal ($100 vs regular cost of $1500)^51^ and CMAS Journal (total waiver of regular cost of $1500 for invited reviews)^52^ if the author is a CF member in good standing.^53^ These benefits are provided to all members who pay a $100 annual membership fee, illustrating a potential significant benefit for members who desire to publish OA. Ultimately, it is paramount that these funding opportunities are equitable to prevent worsening of the disparities that have historically plagued academic medicine and biomedical research.

To our knowledge, this study is the first to explore APCs and potential associations in pathology-related biomedical journals. However, this investigation has some methodologic limitations. Although we used a comprehensive set of pathology journals obtained from a reputable online database, we do not assert that this list is exhaustive, as some pathology subspecialty-specific journals were not present in the database, and we acknowledge that pathologists may publish outside of “traditional” pathology journals. Nevertheless, our utilization of this database minimizes the potential for selection bias. Additionally, while our study is the first to quantify APCs in pathology, it only details current prices, and future studies are necessary to ascertain financial trends. Similarly, we only evaluated a single year's IF for each journal in the multivariable analysis. Finally, certain data points, particularly subscription costs, were not available for a subset of journals, and this lack of data hindered out ability to include these variables in the multivariable analysis.

In summary, this study delineates variables that are significantly associated with pathology journal APCs; however, further research is needed to determine if and how these variables impact the submission and publication process in pathology. These future studies will be valuable in understanding the complex considerations that authors face when attempting to publish scientific manuscripts, and mechanisms by which APCs, OA models, and dissemination of scientific knowledge are interconnected. Additional insight into this process is also crucial in ensuring continued improvement of inequities in academic pathology and biomedical research.

## Disclosure

OF serves as the editor-in-chief of Annals of Diagnostic Pathology, a hybrid journal published by Elsevier. All authors report no relevant conflicts of interests.

## Funding

The authors received no funding for this research.

## Declaration of competing interest

The authors declare that they have no known competing financial interests or personal relationships that could have appeared to influence the work reported in this paper.
