# Supplementary material for: Paying to publish: A cross-sectional analysis of article processing charges and journal characteristics among 87 pathology journals
Source: Acad Pathol. 2024 Nov 20;11(4):100153. doi: 10.1016/j.acpath.2024.100153 (PMC11617391; doi:10.1016/j.acpath.2024.100153)
Supplement: Multimedia component 1 [file mmc1.docx]

**Supplemental Table 1.** Pathology journals included in the analysis.

| **Journal Name** |
| --- |
| Academic Pathology |
| Acta Cytologica |
| Acta Neuropathologica |
| Advances in Anatomic Pathology |
| AJSP-Reviews and Reports |
| Alzheimer Disease & Associated Disorders |
| American Journal of Clinical Pathology |
| American Journal of Forensic Medicine and Pathology |
| American Journal of Pathology |
| American Journal of Surgical Pathology |
| Analytical Cellular Pathology |
| Annales de Pathologie |
| Annals of Diagnostic Pathology |
| Annual Review of Pathology-Mechanisms of Disease |
| APMIS |
| Applied Immunohistochemistry & Molecular Morphology |
| Archives of Pathology & Laboratory Medicine |
| Brain Pathology |
| Brain Tumor Pathology |
| Cancer Cytopathology |
| Cardiovascular Pathology |
| Case Reports in Pathology |
| Cellular Oncology |
| Clinical Neuropathology |
| Clinical Pathology |
| Cytojournal |
| Cytometry Part B-Clinical Cytometry |
| Cytopathology |
| Diagnostic Cytopathology |
| Diagnostic Pathology |
| Disease Models & Mechanisms |
| Endocrine Pathology |
| Experimental and Molecular Pathology |
| Expert Review of Molecular Diagnostics |
| Fetal and Pediatric Pathology |
| Folia Neuropathologica |
| Forensic Science Medicine and Pathology |
| Head & Neck Pathology |
| Histology and Histopathology |
| Histopathology |
| HLA |
| Human Pathology |
| Indian Journal of Pathology and Microbiology |
| International Journal of Clinical and Experimental Pathology |
| International Journal of Experimental Pathology |
| International Journal of Gynecological Pathology |
| International Journal of Immunopathology and Pharmacology |
| International Journal of Paleopathology |
| International Journal of Surgical Pathology |
| Journal of Clinical Pathology |
| Journal of Comparative Pathology |
| Journal of Cutaneous Pathology |
| Journal of Hematopathology |
| Journal of Molecular Diagnostics |
| Journal of Neuropathology And Experimental Neurology |
| Journal of Oral Pathology & Medicine |
| Journal of Pathology |
| Journal of Pathology And Translational Medicine |
| Journal of Pathology Clinical Research |
| Journal of Toxicologic Pathology |
| Laboratory Investigation |
| Leprosy Review |
| Malaysian Journal of Pathology |
| Medecine Nucleaire-Imagerie Fonctionnelle et Metabolique |
| Medical Molecular Morphology |
| Modern Pathology |
| Neuropathology |
| Neuropathology and Applied Neurobiology |
| Pathobiology |
| Pathologia |
| Pathologica |
| Pathologie |
| Pathology |
| Pathology & Oncology Research |
| Pathology and Laboratory Medicine International |
| Pathology International |
| Pathology Research and Practice |
| Pediatric and Developmental Pathology |
| Polish Journal of Pathology |
| Science & Justice |
| Seminars in Diagnostic Pathology |
| Seminars in Immunopathology |
| Toxicologic Pathology |
| Turkish Journal of Pathology |
| Ultrastructural Pathology |
| Veterinary Pathology |
| Virchows Archiv |
